# miR-98-5p, miR-130a-3p, and miR-1246 Profiling in Tissue and Liquid Biopsies of Patients with Hepatocellular Carcinoma

**DOI:** 10.3390/cancers18203379

**Published:** 2026-10-19

**Authors:** Martina Maffezzoni, Ilaria Grossi, Iulia Andreea Pelisenco, Chiara Magri, Sarah Molfino, Giovanni Battista Contessi, Nazario Portolani, Giuseppina De Petro, Flora Guerra, Alessandro Salvi

**Affiliations:** 1 Department of Molecular and Translational Medicine, Università di Brescia, Viale Europa 11, 25123 Brescia, Italy; martina.maffezzoni@unibs.it (M.M.); ilaria.grossi@unibs.it (I.G.); i.pelisenco@unibs.it (I.A.P.); chiara.magri@unibs.it (C.M.); giuseppina.depetro@unibs.it (G.D.P.); 2 Department of Clinical and Experimental Sciences, Università di Brescia, Viale Europa 11, 25123 Brescia, Italy; sarah.molfino@unibs.it (S.M.); nazario.portolani@unibs.it (N.P.); 3 Hepatology Unit, ASST Spedali Civili of Brescia, Piazzale Spedali Civili 1, 25123 Brescia, Italy; giovannibattista.contessi@asst-spedalicivili.it; 4 Department of Biological and Environmental Sciences and Technologies, University of Salento, Via Provinciale Lecce-Monteroni 165, 73100 Lecce, Italy; flora.guerra@unisalento.it

**Keywords:** hepatocellular carcinoma, extracellular vesicles, miRNAs, liquid biopsy, biomarkers

## Abstract

**Background/Objectives**: Hepatocellular carcinoma (HCC) is the most common primary liver malignancy, often arising in cirrhosis. Its early non-invasive diagnosis and prognostic assessment remain challenging due to the limited availability of reliable circulating biomarkers, creating the need to establish non-invasive biomarkers that distinguish malignant transformation. This preliminary study aimed to: discover EV-associated miRNAs that differ among healthy subjects, patients with liver cirrhosis, and patients with HCC; validate selected candidates by ddPCR in an independent cohort; compare their abundance in plasma-derived EVs, whole plasma, and paired tumoral/peritumoral tissues; and explore their diagnostic performance for HCC versus cirrhosis and their associations with clinical outcomes. **Methods**: miRNA sequencing was performed on plasma-derived EVs from an exploratory cohort of 10 healthy subjects, 10 cirrhotic patients, and 10 HCC patients. Three candidate miRNAs, miR-98-5p, miR-130a-3p, and miR-1246, were subsequently quantified by droplet digital PCR (ddPCR) in an independent cohort of 29 HCC patients, 30 cirrhotic patients, and 20 healthy subjects using plasma-derived EVs, plasma, and paired tumoral/peritumoral liver tissues. **Results**: In plasma EVs, miR-98-5p and miR-130a-3p were reduced in HCC and cirrhotic patients compared with healthy subjects, whereas miR-1246 was increased in HCC compared with cirrhosis. In plasma, all three miRNAs were elevated in HCC compared with cirrhosis and healthy controls. Plasma miR-1246 showed the highest diagnostic performance to discriminate HCC from cirrhosis (AUC = 0.87). In tissue, miR-98-5p was upregulated and miR-130a-3p downregulated in tumoral versus peritumoral samples, whereas miR-1246 showed no significant difference. Higher plasma miR-130a-3p and miR-1246 levels were associated with improved overall survival, while increased miR-98-5p levels were associated with lower relapse rates. **Conclusions**: Among the three miRNAs, plasma miR-1246 showed the strongest diagnostic performance for distinguishing HCC from liver cirrhosis. Combination with miR-98-5p and/or miR-130a-3p did not improve discrimination. Thus, plasma miR-1246 emerged as the leading candidate diagnostic indicator in the HCC cohort, whereas the three miRNAs showed distinct potential associations with prognosis.

## Simple Summary

Hepatocellular carcinoma (HCC) often develops in the context of chronic liver disease, and early diagnosis remains challenging. Circulating biomarkers could improve early HCC detection and provide prognostic information. The main aim of our study was to profile miRNAs with translational potential for HCC. Initially, we performed miRNA sequencing on EVs isolated from plasma of healthy individuals, patients with liver cirrhosis, and patients with HCC. Three candidate miRNAs, miR-98-5p, miR-130a-3p, and miR-1246, were subsequently quantified by droplet digital PCR in plasma, tissue, and plasma-derived EVs from an independent cohort. All three miRNAs showed altered levels in HCC patients, with plasma miR-1246 providing the greatest ability to discriminate HCC from liver cirrhosis. Higher plasma levels of all three miRNAs were associated with more favorable clinical outcomes. These exploratory findings highlight circulating miRNAs as both promising candidate indicators for HCC early diagnosis in high-risk patients and as putative HCC prognostic biomolecules.

## 1. Introduction

Hepatocellular carcinoma (HCC) is the most common primary malignant tumor of the liver, ranking as the third leading cause of cancer-related mortality and the sixth most frequently diagnosed cancer worldwide. Its major risk factors include chronic HBV and HCV viral infection, metabolic dysfunction, excessive alcohol consumption and toxin-induced liver injury.

Overall, approximately 90% of HCC cases arise in the context of chronic liver disease, with cirrhosis representing the most significant predisposing condition [1]. Accordingly, current guidelines from the European Association for the Study of the Liver (EASL) and the American Association for the Study of Liver Diseases (AASLD) recommend surveillance with ultrasound (US) every six months, with or without alpha-fetoprotein (AFP) depending on the guideline, for all patients with cirrhosis regardless of etiology. Surveillance is also recommended for individuals with chronic viral hepatitis and for those with MASLD or ALD who meet specific eligibility criteria. Prevention strategies mainly rely on HBV vaccination and antiviral therapy for HBV and HCV infection, and long-term follow-up is recommended to monitor disease progression and residual HCC risk in treated patients [2,3].

Therapeutic options for HCC depend on the patient’s clinicopathological characteristics. Surgical resection is the preferred option for eligible patients and, together with liver transplantation, represents the only potentially curative strategy for early-stage disease. Additional approaches include loco-regional treatment for intermediate-stage HCC and systemic therapies for advanced tumors [1,3].

Despite advances in management, early and non-invasive diagnosis of HCC, as well as accurate prognostic assessment, remain challenging. Liver US and AFP remain the gold-standard modalities for early detection [4]. Additional imaging modalities include contrast-enhanced magnetic resonance imaging (MRI), computed tomography (CT), and contrast-enhanced ultrasound (CEUS). When imaging findings are inconclusive or non-invasive diagnostic criteria are not fulfilled, liver biopsy with histological examination is recommended for diagnostic confirmation, although it is invasive and carries a risk of complications [3]. AFP, measured in serum or plasma, has limited sensitivity and specificity, particularly in early-stage tumors. The establishment of biomarkers is crucial not only for early detection but also for monitoring disease progression, predicting therapeutic response, and identifying potential therapeutic targets [5].

In recent years, growing interest has focused on microRNAs (miRNAs) as diagnostic biomarkers [6], particularly when encapsulated within extracellular vesicles (EVs), which protect their cargo and enable long-distance communication [7,8].

miRNAs are endogenous, small non-coding RNAs of 20–25 nucleotides that regulate the expression of target genes. In cancer, they can act as tumor suppressors when inhibiting protein-coding genes with oncogenic functions, or as oncomiRs when promoting the modulation of tumor-suppressor proteins [9]. miRNAs are released into body fluids through multiple mechanisms, including active secretion *via* exosomes, microvesicles, and protein-mediated export. When not encapsulated within EVs, their association with carrier proteins protects them from nuclease and protease degradation [10].

Focusing specifically on liver malignancies, several circulating miRNAs have also shown diagnostic relevance in HCC [6]. Among these, miR-21 has been reported to be significantly higher in the serum of HCC patients compared with those with liver cirrhosis, supporting its potential as a diagnostic biomarker [11]. Serum miR-122, a liver-specific miRNA, has likewise emerged as a promising diagnostic marker, as it is significantly upregulated in HCC patients compared with healthy subjects [12]. A plasma-based study further indicated its prognostic relevance, showing that higher circulating levels were associated with reduced overall survival (OS) [13]. Several other circulating miRNAs have also been investigated for their diagnostic and prognostic potential in HCC. For example, miR-23b-3p has been reported to be significantly downregulated in untreated patients with HCC compared with healthy subjects and has shown potential as a diagnostic biomarker [14], whereas reduced levels of miR-26a-3p and miR-29a have been associated with worse disease-free survival (DFS) [15].

EVs are delimited by a phospholipid bilayer membrane [16] and are commonly classified according to their biogenesis and size, including exosomes (50–200 nm), microvesicles (100–1000 nm), and apoptotic bodies (50–4000 nm). Virtually all cell types secrete EVs, which are detectable in multiple biological fluids, such as blood, urine, breast milk, saliva, and cerebrospinal fluid [8]. Carrying proteins, nucleic acids, and other non-coding RNAs (ncRNAs), EVs can be internalized by adjacent cells or transported to distant sites, thereby contributing to intercellular communication and modulating diverse cellular activities [17]. In cancer, EVs participate in several key processes including tumor development, angiogenesis, pre-metastatic niche formation, cancer progression, and metastatic dissemination [16,18]. In colorectal cancer (CRC), a recent study reported that miR-125a-3p encapsulated in plasma-derived EVs is upregulated in patients with early-stage disease, supporting its potential as a non-invasive diagnostic biomarker [19]. In HCC, elevated levels of miR-19-3p within circulating plasma EVs have been associated with poorer overall survival, further highlighting the prognostic relevance of EV-derived miRNAs [15]. Similarly, elevated plasma EV-associated miR-92b levels have been linked to an increased risk of early recurrence following liver transplantation [20].

The first aim of this study was to identify circulating miRNAs as potential non-invasive biomarkers for HCC. To this end, we initially performed miRNA-sequencing (miRNA-seq) on EVs isolated from plasma samples of a discovery cohort including healthy subjects, cirrhotic patients, and HCC patients. To investigate the relevance of specific ncRNAs in HCC, we examined selected miRNA candidates and quantified their levels by droplet digital PCR (ddPCR) in a larger cohort and across multiple biological matrices.

## 2. Materials and Methods

### 2.1. Participants

For the present study, a total of 109 participants were included and divided into two cohorts: a discovery and a testing cohort. The discovery cohort consisted of 10 healthy subjects (HS), 10 patients with liver cirrhosis (LC), and 10 patients with HCC (HCC), whereas the testing cohort comprised 20 HS, 30 patients with LC, and 29 with HCC. Peripheral blood was collected from all participants into EDTA-containing blood collection tubes. Plasma was separated within 30 min of collection using the same pre-analytical protocol for all study participants. Briefly, blood samples were first centrifuged at 3000 rpm for 10 min at 4 °C, followed by a second centrifugation at 4000 rpm for 20 min at 4 °C to obtain cell-free plasma, as previously described [14]. Following plasma separation, samples were visually inspected for pink or red discoloration suggestive of overt hemolysis. None of the samples included in the study showed visible evidence of hemolysis. A quantitative spectrophotometric or miRNA-based hemolysis index was not available. Plasma was stored at −80 °C. In HCC treatment-naïve patients, peripheral blood samples were collected prior to surgery. In addition, both tumoral and peritumoral tissue samples were collected from HCC patients and preserved in RNA*later* Stabilization Solution (Invitrogen, Thermo Fisher Scientific, catalog no. AM7020, Waltham, MA, USA), which were stored at −20 °C for subsequent RNA extraction. The main clinicopathological characteristics of HCC patients were recorded and are reported in Table 1. Table S1 reports the clinicopathological characteristics of HCC patients stratified according to cirrhosis as the underlying liver disease. Table S2 summarizes the clinicopathological characteristics of HS and LC. AFP levels were not reported for LC because these data were available for only a very small proportion of the cohort.

This study was approved by the Ethics Committee of the ASST Spedali Civili, Brescia, Italy (study reference number NP 5291, 7 June 2022, and amended on 14 May 2024). All participants provided written informed consent prior to enrollment.

### 2.2. RNA Purification and Reverse Transcription (RT)

RNA associated with a membrane-affinity-captured extracellular-particle fraction was isolated from 1 mL of plasma using the exoRNeasy Midi Kit (Qiagen, catalog no. 77144, Hilden, Germany), according to the manufacturer’s RNA-isolation protocol. Plasma was loaded onto the exoEasy membrane-affinity column. The retained material was lysed directly on the column using QIAzol Lysis Reagent. Following chloroform-induced phase separation, RNA contained in the aqueous phase was purified using an RNeasy MinElute spin column and eluted in RNase-free water. Because the retained particles were directly lysed rather than eluted intact with Buffer XE, EV concentration, size distribution, morphology, protein markers, and purity could not be assessed on the material used for RNA analysis.

Circulating RNAs derived from plasma were obtained using the miRNeasy Serum/Plasma Advanced Kit (Qiagen, catalog no. 217204), following the manufacturer’s instructions. Total RNA from tissue biopsies was extracted using TRIzol reagent (Thermo Fisher Scientific, catalog no. 15596026, Waltham, MA, USA), as recommended by the manufacturer, and subsequently quantified using Nanodrop ND-100 (Thermo Fisher Scientific).

For tissue biopsy analysis, cDNA of the selected miRNAs was synthesized from 50 ng of total RNA using the miRCURY LNA RT Kit (Qiagen, catalog no. 339340, Hilden, Germany), following the manufacturer’s instructions. For plasmatic RNA and plasma-derived EVs, cDNA of the selected miRNAs was synthesized from 1.5 μL of total RNA using the miRCURY LNA RT Kit (Qiagen). cDNA synthesis was performed in a final reaction volume of 10 μL, containing 2 μL of 5× miRCURY RT SYBR Green Reaction Buffer, 1 μL of 10× miRCURY RT Enzyme mix, and 0.5 μL of UniSP6 RNA Spike-in template; RNase-free water was added to reach the final volume. The thermal profile consisted of incubation at 42 °C for 60 min followed by enzyme inactivation at 95 °C for 5 min.

### 2.3. Small RNA Sequencing

Library preparation and sequencing were performed as a service by NGS Biodiversa s.r.l. (Treviso, Italy), on RNA extracted from plasma-derived EVs of the 30 samples of the discovery cohort. Briefly, small RNA libraries were generated using the SMARTer smRNA-Seq Kit (Takara Bio Inc., catalog no. 635029, Shiga, Japan), following the manufacturer’s protocol. The libraries were subsequently sequenced on a NovaSeq 6000 (Illumina, catalog no. 20012850, San Diego, CA, USA) using the NovaSeq 6000 SP Reagent Kit (Illumina, catalog no. 20028400), producing paired-end reads of 150 nucleotides. Small RNA sequencing yielded a high-quality dataset with an average of 15.3 million total input reads per sample, ensuring adequate coverage for the detection and quantification of low-abundance miRNAs. For downstream analysis, only the R1 reads were retained. R1 was selected as the primary analytical read because it provides sufficient sequence information to cover the small RNA insert and enable adapter trimming, sequence identification, and miRNA annotation.

Adapter trimming, alignment, and miRNA quantification were performed using miRge3.0 [21]. Trimming parameters were set according to the SMARTer smRNA-Seq protocol instruction. Specifically, the first three nucleotides at the 5′ end of each read were removed, together with the 3′ poly(A) stretch, the adapter sequence, and any downstream bases, including residual Illumina adapter sequences. Processed reads were then collapsed and aligned to the human miRNA reference database from miRBase using the built-in libraries provided with miRge3.0. miRNA expression levels were quantified as the number of reads mapped to each annotated miRNA, generating count tables for each sample suitable for downstream analyses. One LC sample failed during library preparation and was therefore not sequenced. The annotation process identified a consistent repertoire of miRNAs across the dataset, with an average of 113,878 filtered miRNA reads per library. On average, a mean of 65 unique miRNA species was identified per sample. The raw miRNA-seq data have been deposited in the Sequence Read Archive (SRA) under accession number PRJNA1525574.

### 2.4. Differential Expression Analysis of miRNAs

miRNA differential expression analysis (miDEA) was performed using the DESeq2 package in R, as described in Love et al., 2014 [22]. Principal Component Analysis (PCA) was performed with the PlotPCA function implemented in the DESeq2 package on the regularized log-transformed (rlog) data, using the totality of miRNA analyzed. Wald test implemented in DESeq2 was used to identify differentially expressed miRNA (miRDEG). Only miRNA expressed with at least 20 reads in at least five samples were considered for the analysis. False discovery rate BH FDR was used to control for multiple testing comparisons. Differentially expressed miRNAs were defined as padj < 0.05 and log_2_ fold change (log_2_FC) ≤ −1 or ≥1. Volcano plots and the heatmap were generated using ggplot2 and pheatmap packages, respectively [23,24]. miRNAs showing statistically significant differences in at least one comparison were included in the heatmap, which was constructed using raw log_2_FC values. These statistical analyses were performed in RStudio v2025.05.01+513 running R v4.5.1 [25,26].

### 2.5. Absolute Quantification of miR-98-5p, miR-130a-3p, and miR-1246 Using ddPCR

The resulting cDNA was diluted to the appropriate concentration and then used as a template for the droplet digital PCR (ddPCR) experiment performed with the QX200 ddPCR System (Bio-Rad Laboratories, Hercules, CA, USA). ddPCR reactions were prepared according to the QX200 ddPCR EvaGreen Supermix (Bio-Rad, catalog no. 186-4033, Hercules, CA, USA) protocol. Specifically, 8 μL of cDNA were amplified in a final reaction volume of 20 μL containing 2× QX200 ddPCR EvaGreen Supermix (Bio-Rad) and the appropriate miRCURY miRNA PCR Assay primers (Qiagen, cat. n. 339306) at a volume of 1.1 μL: miR-98-5p (Qiagen, Assay ID: YP00204640), miR-130a-3p (Qiagen, Assay ID: YP00204658), and miR-1246 (Qiagen, Assay ID: YP00205630). Each reaction mixture was loaded into DG8 Cartridges (Bio-Rad, catalog no. 1864008), and 70 μL of QX200 Droplet Generation Oil for EvaGreen (Bio-Rad, catalog no. 1864005) was added to the designated wells. After droplet generation, the droplets were transferred into a 96-well plate (Bio-Rad, 12001925), which was then heat-sealed with the PCR Plate Heat Seal foil (Bio-Rad, catalog no. 1814040) and placed into a T100 Thermal Cycler (Bio-Rad, catalog no. 1861096). As the 60 °C annealing temperature recommended for EvaGreen assays was not optimal for our primer sets, annealing temperatures were experimentally optimized and set to 58 °C for miR-98-5p and miR-1246, and to 56 °C for miR-130a-3p. The concentrations of miR-98-5p, miR-130a-3p, and miR-1246 were quantified using QuantaSoft Software v1.7 (Bio-Rad) and expressed as copies/μL. We performed two independent ddPCR experiments to assess technical and biological reproducibility. ddPCR provides absolute quantification by partitioning each 20 µL reaction into approximately 20,000 droplets, thereby improving analytical precision and sensitivity. The repeat experiment confirmed the reproducibility of the measurements; the ddPCR results presented refer to one representative experiment.

### 2.6. Statistical Analysis

Statistical analyses were conducted using GraphPad Prism v10.3.1 [27].

Normality of distributions was assessed using the Shapiro–Wilk test to determine whether parametric or non-parametric tests were appropriate. Differences in miRNA levels in plasma-derived EVs and plasma were assessed using the Mann–Whitney U test, and analyses for these biological matrices were additionally stratified according to clinicopathological characteristics. Differences in miR-98-5p and miR-1246 levels between relapsed and non-relapsed patients were evaluated using the Mann–Whitney U test due to the non-normal distribution of the data. Differences in plasma miR-130a-3p levels between patients with G2 and G3 tumors were assessed using the unpaired Student’s *t*-test, as these data followed a normal distribution. For miRNA expression levels in solid biopsies, values were log_10_-transformed due to their non-normal distribution. Paired differences between log-transformed PT and HCC values were then computed, and normality of these paired differences was confirmed using the Shapiro–Wilk test before applying paired Student’s *t*-test. To assess the potential diagnostic performance of selected miRNAs, Receiver Operating Characteristic (ROC) curve analyses were performed using the pROC package [24]. The area under the curve (AUC) and corresponding 95% confidence intervals (95% CI) were calculated using non-parametric methods, and statistical significance was assessed against the null hypothesis of AUC = 0.5. Optimal cutoff values were determined using the Youden index, and sensitivity and specificity were calculated accordingly. To evaluate whether combinations of miRNAs improved diagnostic performance, multivariable logistic regression models were constructed using generalized linear models with a binomial distribution. Predicted probabilities derived from each model were used to generate combined ROC curves, and differences between AUCs were assessed using the DeLong test for correlated ROC curves. All ROC-related analyses were performed in RStudio v2025.05.01+513 running R v4.5.1 [25,26]. For survival analyses, the median value of each miRNA was used as the cutoff: patients with levels greater than the median were classified as having high levels, whereas those with levels equal to or below the median were classified as having low levels. This classification was applied to both plasma and plasma-derived EVs. Overall survival (OS) and disease-free survival (DFS) were evaluated using the Mantel–Cox test. These analyses were conducted using GraphPad Prism v10.3.1 [27].

### 2.7. miRNA Target Gene Prediction and Functional Enrichment Analysis

Three databases were used to predict gene targets for each miRNA, including TargetScan (release 8.0) [28], miRWALK (version 3.0) [29], and miRDIP [30] or MicroT DIANA [31,32] for miR-1246 only. To refine the results, the following filtering criteria were applied: in miRDIP, the score class was set to ‘Very high’ and ‘high’, and in miRWALK, the position was set to ‘3′UTR’. The resulting predicted targets common to the databases were then subjected to Gene Ontology (GO) enrichment analysis using the DAVID website [33,34] to investigate their associated biological processes, molecular functions and cellular components, as well as to characterize the significantly enriched pathways according to KEGG annotation.

## 3. Results

### 3.1. RNA-Sequencing Analysis on Small RNA Content of Plasma-Derived EVs

**Figure 1:**
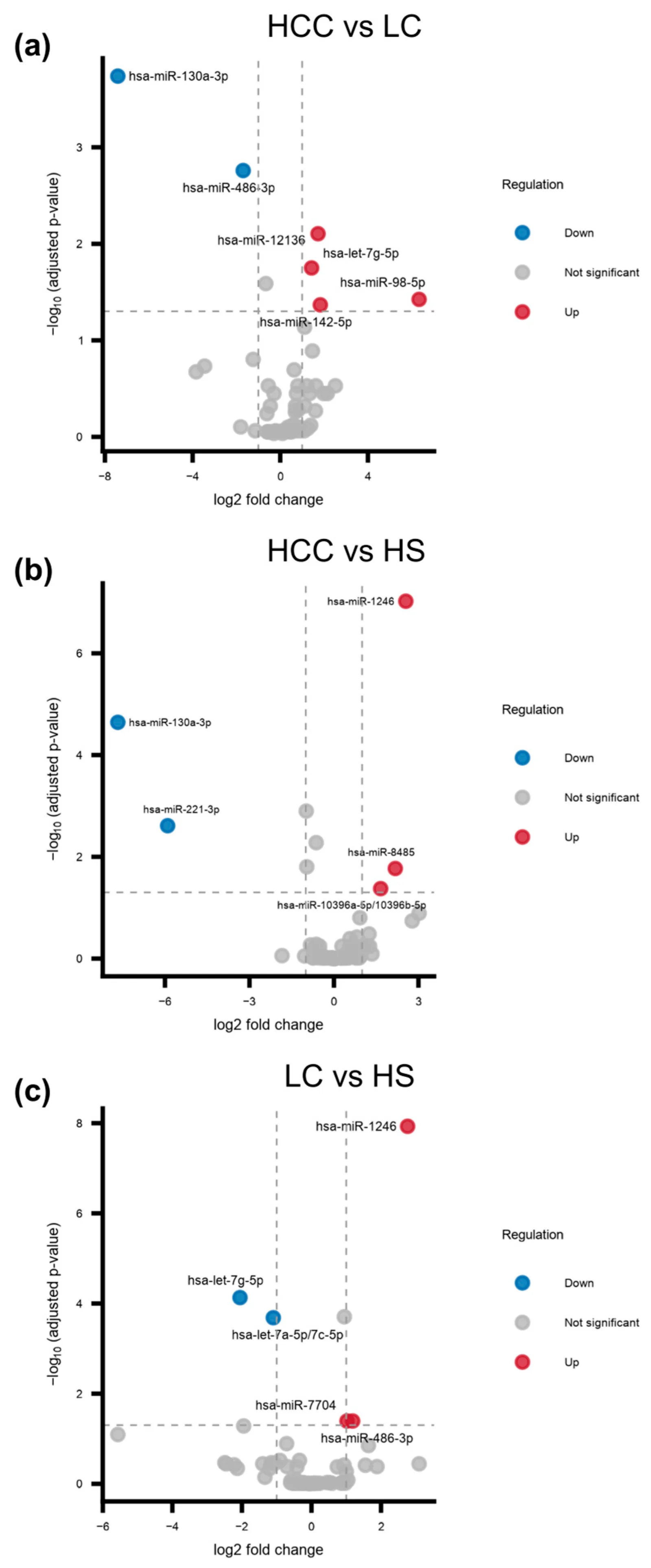
miRNA-sequencing analysis of plasma-derived EVs in the discovery cohort**.** Volcano plots showing miRNAs detected in EVs from HCC and LC (**a**), HCC and HS (**b**), and LC and HS (**c**). miRNAs highlighted in red or blue represent those significantly dysregulated in the specific comparisons shown in each panel. Dashed vertical lines indicate log2 fold change thresholds of −1 and +1, whereas the dashed horizontal line indicates the adjusted *p*-value threshold of 0.05. miRNAs with an adjusted *p*-value < 0.05 and an absolute log2 fold change ≥ 1 were considered differentially expressed. HS: healthy subjects; LC: patients with liver cirrhosis; HCC: patients with HCC.

To profile miRNAs detectable in plasma-derived EVs, miRNA-sequencing was performed in the discovery cohort, which included healthy subjects (HS; *n* = 10), patients with liver cirrhosis (LC; *n* = 10), and patients with HCC (HCC; *n* = 10). A total of 59 miRNAs were listed after applying a filtering threshold of at least 20 reads in a minimum of five samples. To assess the overall similarity between the miRNA expression profiles of the different groups, we performed a Principal Component Analysis (PCA). The first two principal components accounted for 30% of the total variance (PC1: 18%, PC2: 12%). The PCA showed a good separation along the PC1 axis between HS and both LC and HCC, whereas LC and HCC groups displayed some degree of overlap (Figure S1). Differential expression analysis identified distinct miRNA profiles across the study groups (Table S3). Specifically, we found five differentially expressed miRNAs (miRNADEGs) in the comparison between LC and HS. Moreover, five miRNAs were significantly dysregulated in HCC versus HS, while six miRNAs reached statistical significance when comparing HCC to LC. Four miRNAs, highlighted in bold in Table S3, were differentially expressed in more than two comparisons. Based on their distinct expression profiles and statistical significance across the comparisons, we selected miR-98-5p, miR-130a-3p, and miR-1246 for downstream analyses. miR-98-5p was significantly upregulated in EVs of HCC compared with LC (comparison HCC vs. LC: log2FC = 6.34, *p* < 0.01; Figure 1a and Table S3).

In contrast, miR-130a-3p was significantly downregulated in the HCC group compared with LC and HS (log2FC = −7.41, *p* < 0.001; log2FC = −7.67, *p* < 0.0001, respectively; Figure 1a and Table S3). miR-1246 exhibited significant upregulation when either LC (comparison LC vs. HS: log2FC = 2.76, *p* < 0.0001) or HCC (comparison HCC vs. HS: log2FC = 2.55, *p* < 0.0001) are compared with HS (Figure 1, panels b/c, and Table S3). These distinct level patterns were evidenced in the heatmap in Figure S2.

### 3.2. miR-98-5p, miR-130a-3p, and miR-1246 Are Differentially Encapsulated in Plasma-Derived EVs of HCC Patients

**Figure 2:**
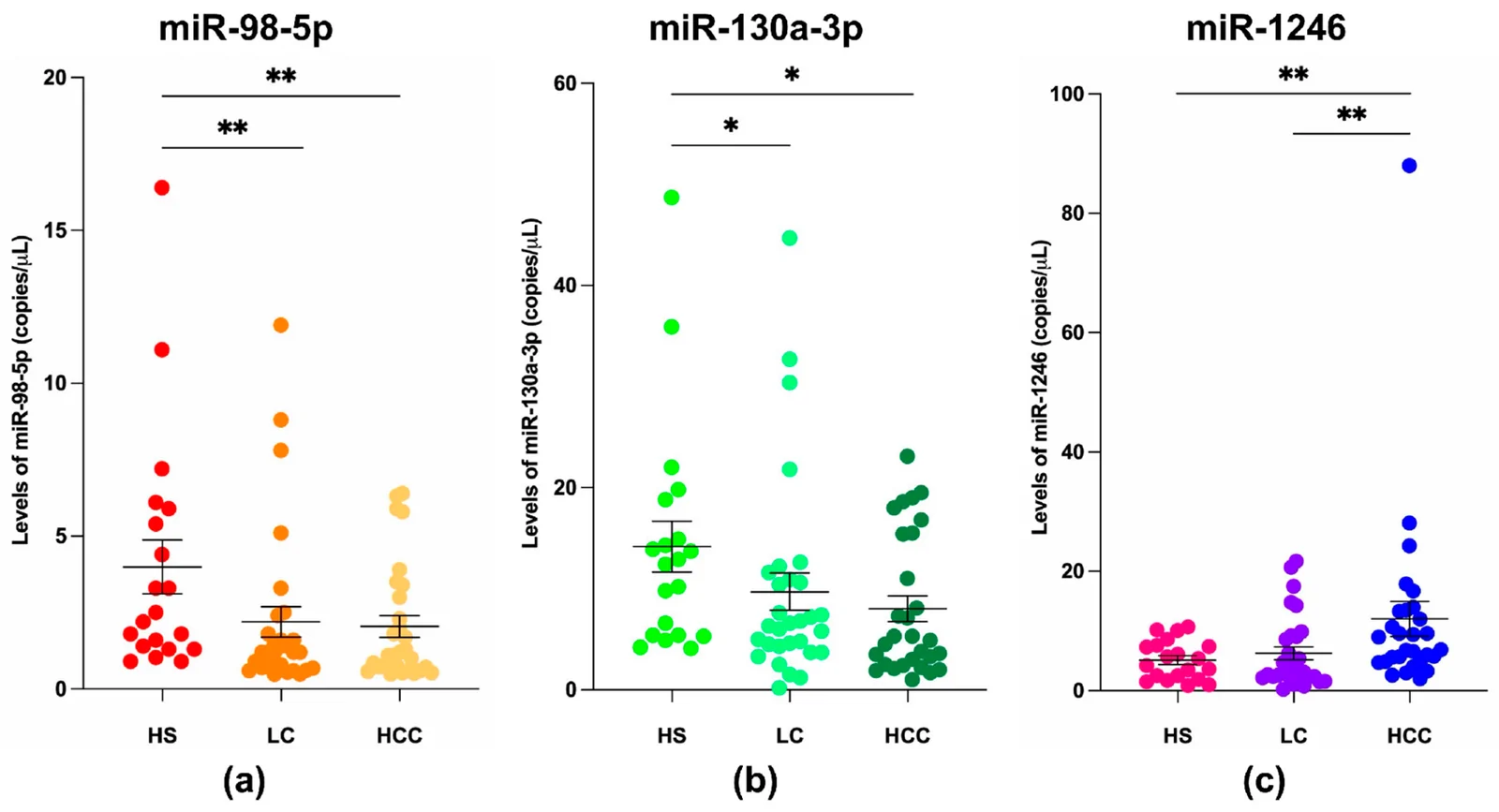
Levels of selected miRNAs detected by ddPCR in plasma-derived EVs from the testing cohort. Dot plots representing the levels of (**a**) miR-98-5p, (**b**) miR-130a-3p, and (**c**) miR-1246 in plasma-derived EVs from the testing cohort. Each graph shows the level of the miRNA in the three study groups. Data are presented as mean ± SEM. Mann–Whitney U test was performed: *, ** mean *p* < 0.05, *p* < 0.01, respectively. HS: healthy subjects; LC: patients with liver cirrhosis; HCC: patients with HCC.

Following the identification in the discovery cohort by miRNA-sequencing analysis, miR-98-5p, miR-130a-3p, and miR-1246 were quantified by ddPCR in plasma-derived EVs from the testing cohort, which comprised HS (*n* = 20), LC (*n* = 30), and HCC (*n* = 29). The mean levels of miR-98-5p were significantly lower in HCC and LC compared with HS (HCC vs. HS: 2.05 vs. 4.00 copies/μL, *p* < 0.01; LC vs. HS: 2.20 vs. 4.00 copies/μL, *p* < 0.01, respectively), with the ratios of 0.51 and 0.55, respectively (Figure 2a).

The mean levels of miR-130a-3p were also significantly lower in HCC and LC compared with HS (HCC vs. HS: 8.01 vs. 14.16 copies/μL, *p* < 0.05; LC vs. HS: 9.70 vs. 14.16 copies/μL, *p* < 0.05), with the ratios of 0.57 and 0.69, respectively (Figure 2b). miR-98-5p and miR-130a-3p showed comparable levels between HCC and LC. miR-1246 (Figure 2c) showed significantly higher levels in EVs from HCC compared with either LC or HS (HCC vs. LC: 12.03 vs. 6.27 copies/μL, *p* < 0.01; HCC vs. HS: 12.03 vs. 5.12 copies/μL, *p* < 0.01), with the ratios of 1.92 and 2.35, respectively.

### 3.3. Plasma Levels of miR-98-5p, miR-130a-3p, and miR-1246 Are Higher in HCC Compared with LC or HS

**Figure 3:**
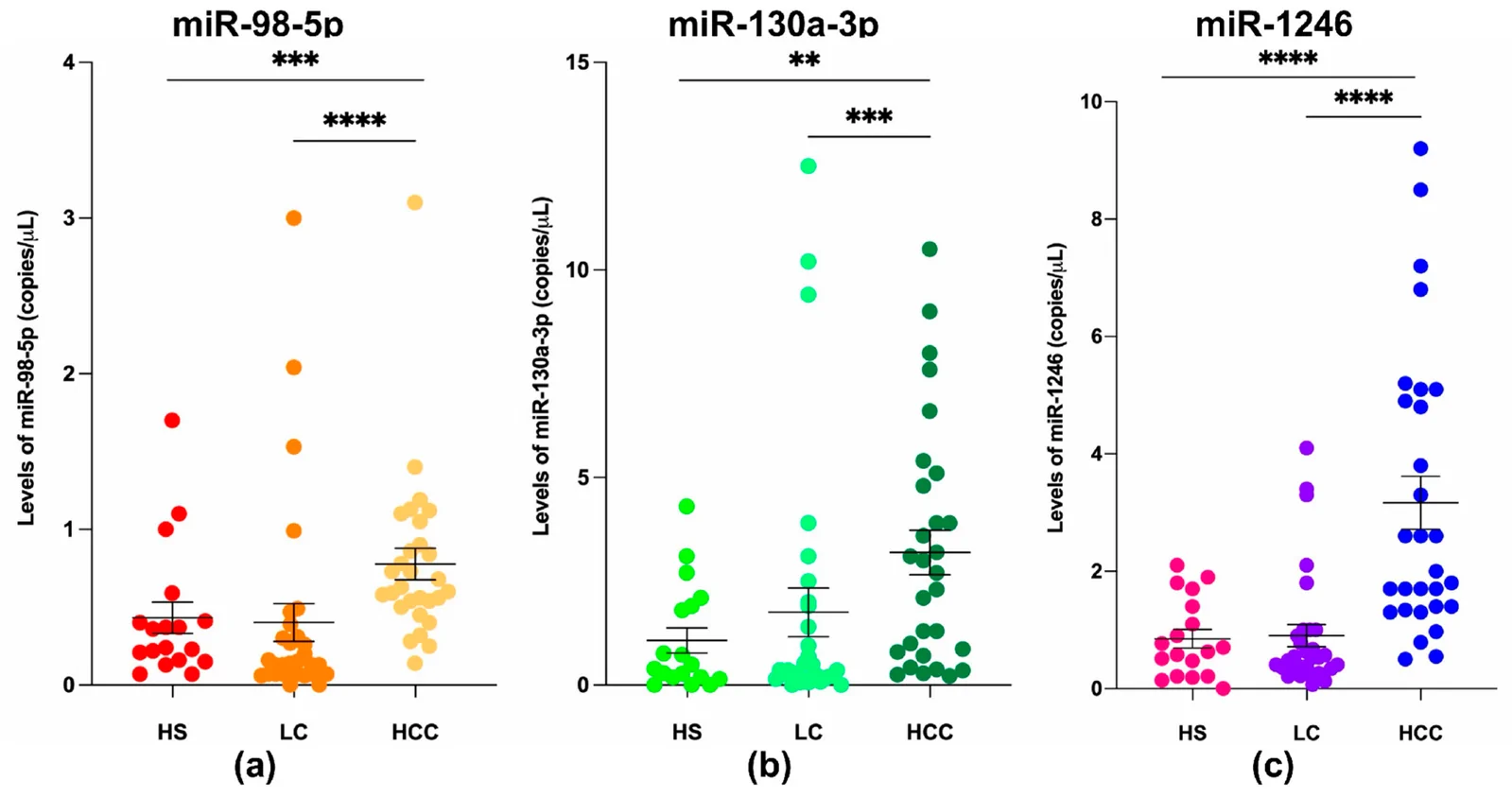
Levels of the selected miRNAs detected by ddPCR in plasma samples from the testing cohort. Dot plots showing the levels of (**a**) miR-98-5p, (**b**) miR-130a-3p, and (**c**) miR-1246 in plasma of the three study groups. Data are presented as mean ± SEM. Statistical significance was assessed using the Mann–Whitney U test. ** = *p* < 0.01, *** = *p* < 0.001, **** = *p* < 0.0001. HS: healthy subjects; LC: patients with liver cirrhosis; HCC: patients with HCC.

After measuring the levels of the selected ncRNAs in plasma EVs, we quantified their corresponding circulating plasma miRNA levels in individuals from the testing cohort. The aim was to assess whether these circulating ncRNAs could discriminate among groups without the need for EV purification, and to compare the two approaches. For miR-98-5p (Figure 3a), plasma levels were significantly higher in HCC compared with LC or HS (HCC vs. LC: 0.78 vs. 0.40 copies/μL, *p* < 0.0001; HCC vs. HS: 0.78 vs. 0.43 copies/μL, *p* < 0.001), with the ratios of 1.95 and 1.81, respectively.

miR-130a-3p (Figure 3b) showed a similar pattern, with significantly higher levels in HCC compared with LC or HS (HCC vs. LC: 3.20 vs. 1.75 copies/μL, *p* < 0.001; HCC vs. HS: 3.20 vs. 1.07 copies/μL, 0.01), with ratios of 1.83 and 2.99, respectively. miR-1246 (Figure 3c) also displayed significantly higher levels in HCC compared with LC or HS (HCC vs. LC: 3.17 vs. 0.9 copies/μL, *p* < 0.0001; HCC vs. HS: 3.17 vs. 0.85 copies/μL, *p* < 0.0001) with the ratios of 3.52 and 3.73, respectively. Notably, plasma miR-1246 exhibited the most relevant increase, with an approximately 4-fold elevation in HCC compared with HS, and this was consistent with the trend observed in EVs.

### 3.4. miR-98-5p and miR-130a-3p Are Dysregulated in HCC Tissues from Biopsy Specimens

**Figure 4:**
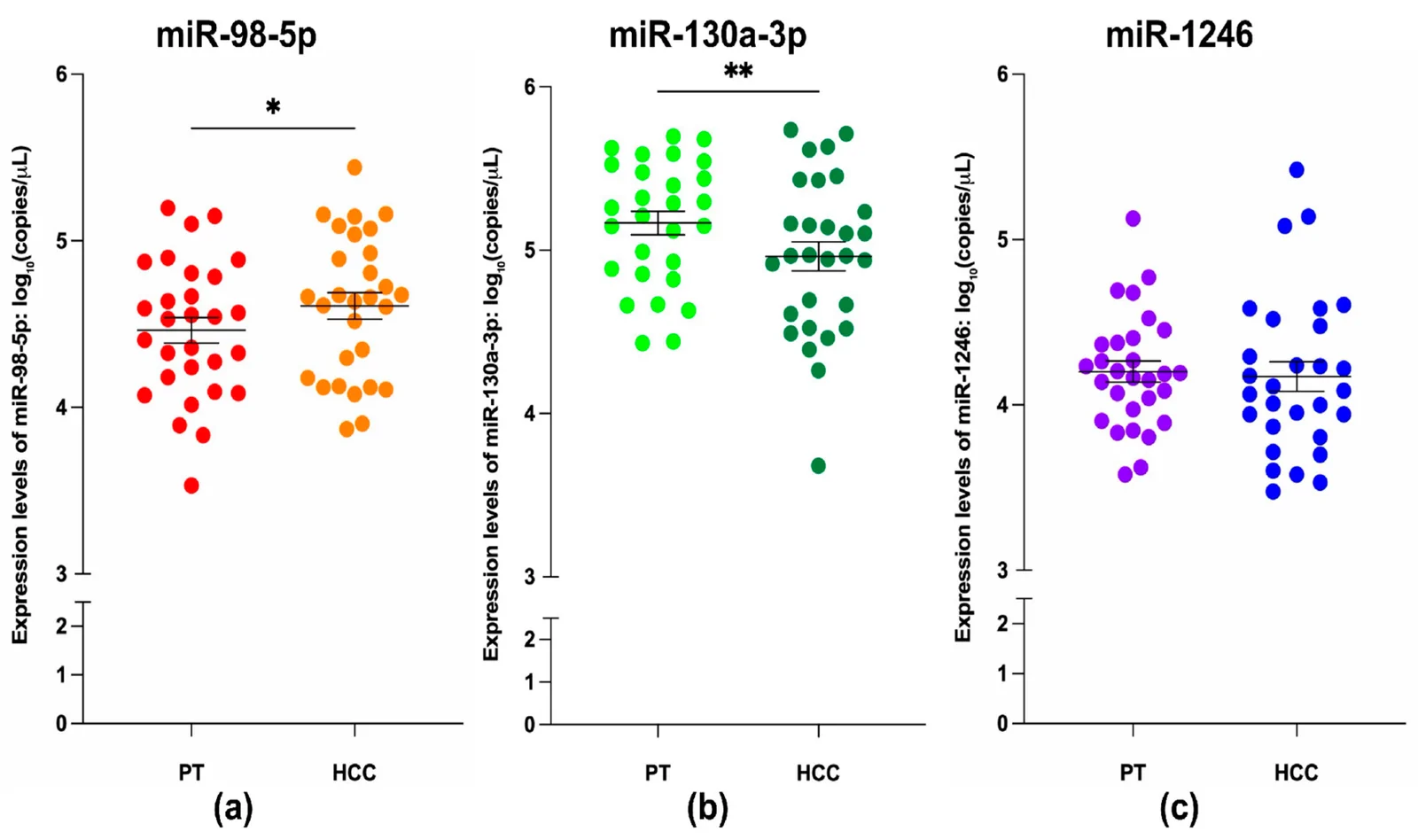
Expression levels of miR-98-5p, miR-130a-3p, and miR-1246 in tissue biopsy from the testing cohort. Dot plots depicting expression levels of (**a**) miR-98-5p, (**b**) miR-130a-3p, and (**c**) miR-1246. Each graph shows the expression levels of miRNA detected in solid biopsy in HCC group. Data are presented as mean ± SEM. Paired *t*-test was performed: * = *p* < 0.05; ** = *p* < 0.01. PT: Peritumoral tissue; HCC: tumoral tissue.

In matched peritumoral (PT) and tumoral (HCC) specimens, the selected miRNAs exhibited two distinct expression patterns. miR-98-5p showed a significant upregulation in HCC compared with the corresponding PT samples (R_HCC/PT_ = 1.39, *p* < 0.05; Figure 4a).

In contrast, miR-130a-3p was markedly downregulated in HCC compared with PT tissues (R_HCC/PT_ = 0.75, *p* < 0.05; Figure 4b), while miR-1246 displayed comparable expression levels in HCC tissues compared with PT (R_HCC/PT_ = 0.93, *p* > 0.05; Figure 4c). Statistical analyses and graphical representations were performed on log-transformed data to account for their log-normal distribution; accordingly, the means reported in the text refer to the averages of the log-transformed datasets. Expression ratios between HCC and PT were then back-transformed to express fold changes on a linear scale and to determine whether each miRNA was up- or downregulated in HCC relative to PT. Notably, among the three miRNAs, miR-1246 was the least abundant in tissue samples—opposite to what was observed in plasma EVs and in the plasma fraction, where it represented the most abundant miRNA.

The ddPCR results for the three selected miRNAs across the three biological matrices are summarized in Table S4.

### 3.5. miR-98-5p, miR-130a-3p, and miR-1246 as Candidate Early Diagnostic Indicators in LC High-Risk Patients

**Figure 5:**
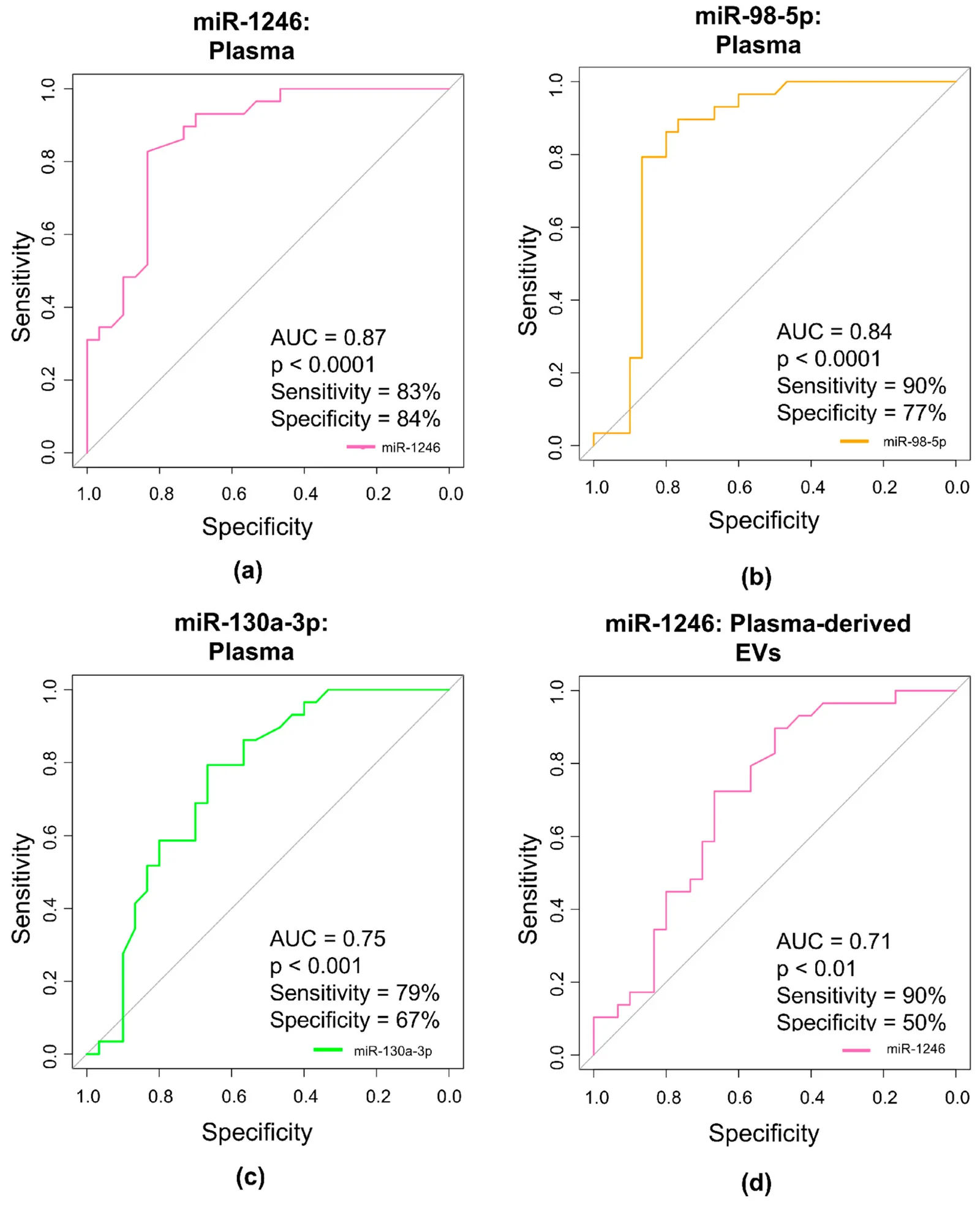
Receiver Operating Characteristic (ROC) curve analysis of plasma and EV-derived miRNAs to discriminate between HCC and liver cirrhosis patients. (**a**) Plasma miR-1246 (AUC = 0.87, *p* < 0.0001); (**b**) plasma miR-98-5p (AUC = 0.84, *p* < 0.0001); (**c**) plasma miR-130a-3p (AUC = 0.75, *p* < 0.001); (**d**) EV-derived miR-1246 (AUC = 0.71, *p* < 0.01).

To evaluate the diagnostic performance of plasma miR-98-5p, miR-130a-3p, and miR-1246, Receiver Operating Characteristic (ROC) curve analyses were performed to assess their ability to distinguish patients with HCC from LC. All three miRNAs demonstrated significant discriminatory ability, with miR-1246 (Figure 5a) showing the highest performance (AUC = 0.87, *p* < 0.0001), indicating good diagnostic accuracy.

At the optimal cutoff value, miR-1246 reached a sensitivity of 82.80% and a specificity of 83.80%, indicating a strong ability to detect true positive cases while keeping the number of incorrect positive classifications low. miR-98-5p (Figure 5b) exhibited a comparable diagnostic accuracy (AUC = 0.84, *p* < 0.0001). Notably, miR-98-5p achieved a higher sensitivity (89.70%) but a lower specificity (77.00%). miR-130a-3p (Figure 5c) showed a small yet statistically significant discriminatory capacity (AUC = 0.75, *p* < 0.001), with a sensitivity of 79.30% and a specificity of 66.70%—both of them lower than those of miR-98-5p and miR-1246 (Table S5). To investigate whether combining miRNAs could enhance diagnostic performance, multivariable logistic regression models were generated. As shown in Table S5, the combination of miR-98-5p and miR-130a-3p did not improve the AUC compared with miR-98-5p alone (AUC = 0.84, *p* < 0.0001). Similarly, combining miR-98-5p and miR-1246 did not yield an AUC higher than that of miR-1246 alone (AUC = 0.87, *p* < 0.0001), nor did the miR-130a-3p/miR-1246 model (AUC = 0.86, *p* < 0.0001) outperform miR-1246 as a single biomarker. Notably, even the three-miRNA combined model (AUC = 0.86, *p* < 0.0001) did not result in any improvement over miR-1246 alone. Overall, none of the combined models resulted in a statistically significant increase in AUC compared with miR-1246 alone. We also assessed the potential diagnostic value of miR-98-5p, miR-130a-3p, and miR-1246 in plasma-derived EVs. Only miR-1246 (Figure 5d) showed a statistically significant ability to discriminate between patients with HCC and those with LC. EV-derived miR-1246 yielded an AUC of 0.71 (*p* < 0.01), with a high sensitivity (89.70%), but limited specificity (50.00%).

To specifically assess the diagnostic performance of the selected miRNAs in the clinically relevant population of HCC arising in cirrhosis, ROC analyses were performed comparing the 16 HCC patients with underlying cirrhosis with the 30 LC patients. miR-98-5p showed an AUC of 0.84, while miR-130a-3p and miR-1246 showed AUCs of 0.75 and 0.87, respectively (Figure S3 and Table S6). To further investigate performance in early-stage disease, the analysis was restricted to the 13 patients with cirrhotic HCC classified as BCLC stage 0 or A. The AUCs were 0.83 for miR-98-5p, 0.79 for miR-130a-3p, and 0.86 for miR-1246 (Figure S4 and Table S6). All of these analyses were statistically significant. Overall, plasma miR-1246 demonstrated the highest diagnostic accuracy among all the models evaluated.

### 3.6. miR-98-5p, miR-130a-3p, and miR-1246 as Non-Invasive Potential Prognostic Biomarkers

**Figure 6:**
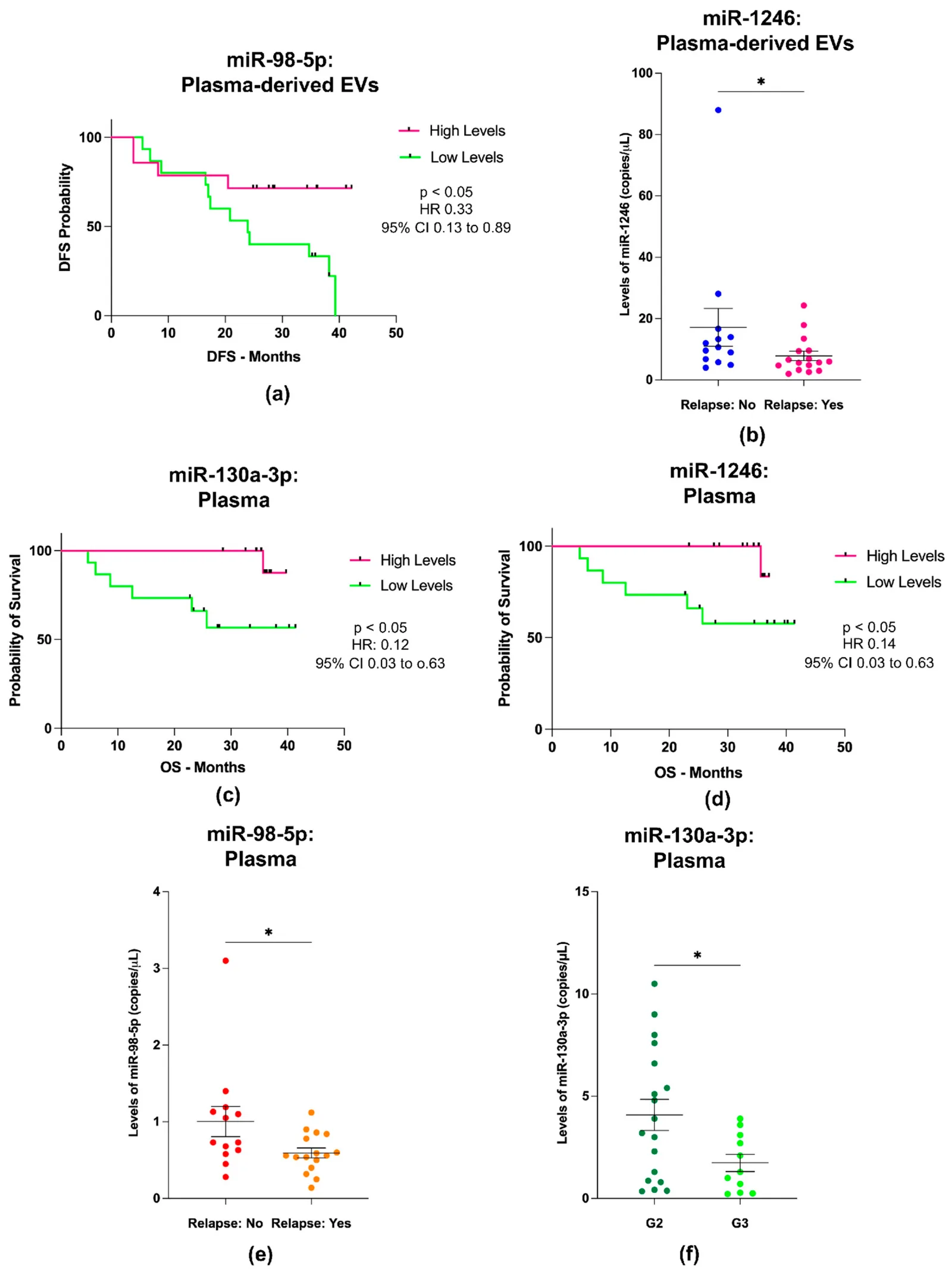
Prognostic significance and clinicopathological associations of plasma and EV-derived miRNAs. (**a**) DFS based on EV-derived miR-98-5p levels. (**b**) EV-derived miR-1246 levels in relation to relapse status. (**c**) OS based on plasma miR-130a-3p levels. (**d**) OS based on plasma miR-1246 levels. (**e**) Plasma miR-98-5p levels in relapsed vs. non-relapsed patients. (**f**) Plasma miR-130a-3p levels according to tumor histological grade. For clinicopathological characteristics, statistical analyses were performed using the Unpaired *t*-test or Mann–Whitney U test, as appropriate. * = *p* < 0.05.

We also evaluated the association of circulating miR-98-5p, miR-130a-3p, and miR-1246 with disease-free survival (DFS) and overall survival (OS) in both plasma and in plasma-derived EVs. High levels of EV-associated miR-98-5p were positively correlated with prolonged DFS (Figure 6a).

In addition, EV-derived miR-1246 concentrations were higher in patients who did not experience relapse compared with those who relapsed (Relapse: No = 17.15 copies/µL vs. Relapse: Yes = 7.87 copies/µL, *p* < 0.05; Figure 6b). Regarding plasma miRNAs, the patients with longer OS displayed higher plasma levels of miR-130a-3p (Figure 6c) and miR-1246 (Figure 6d). Moreover, plasma miR-98-5p levels were significantly higher in patients without relapse compared with those who relapsed (Relapse: No = 1.00 copies/µL vs. Relapse: Yes = 0.59 copies/µL, *p* < 0.05; Figure 6e). Instead, plasma miR-130a-3p concentrations were elevated in patients with histological grade 2 tumors relative to those with grade 3 tumors (G2 = 4.08 copies/µL vs. G3 = 1.74 copies/µL, *p* < 0.05; Figure 6f). No other significant associations were observed between the analyzed miRNAs and the evaluated clinicopathological features or survival outcomes (Table S7).

In light of these findings, the associations with the key prognostic factors should be regarded as promising but still exploratory.

### 3.7. Prediction of Target Genes and GO Enrichment Analysis

**Figure 7:**
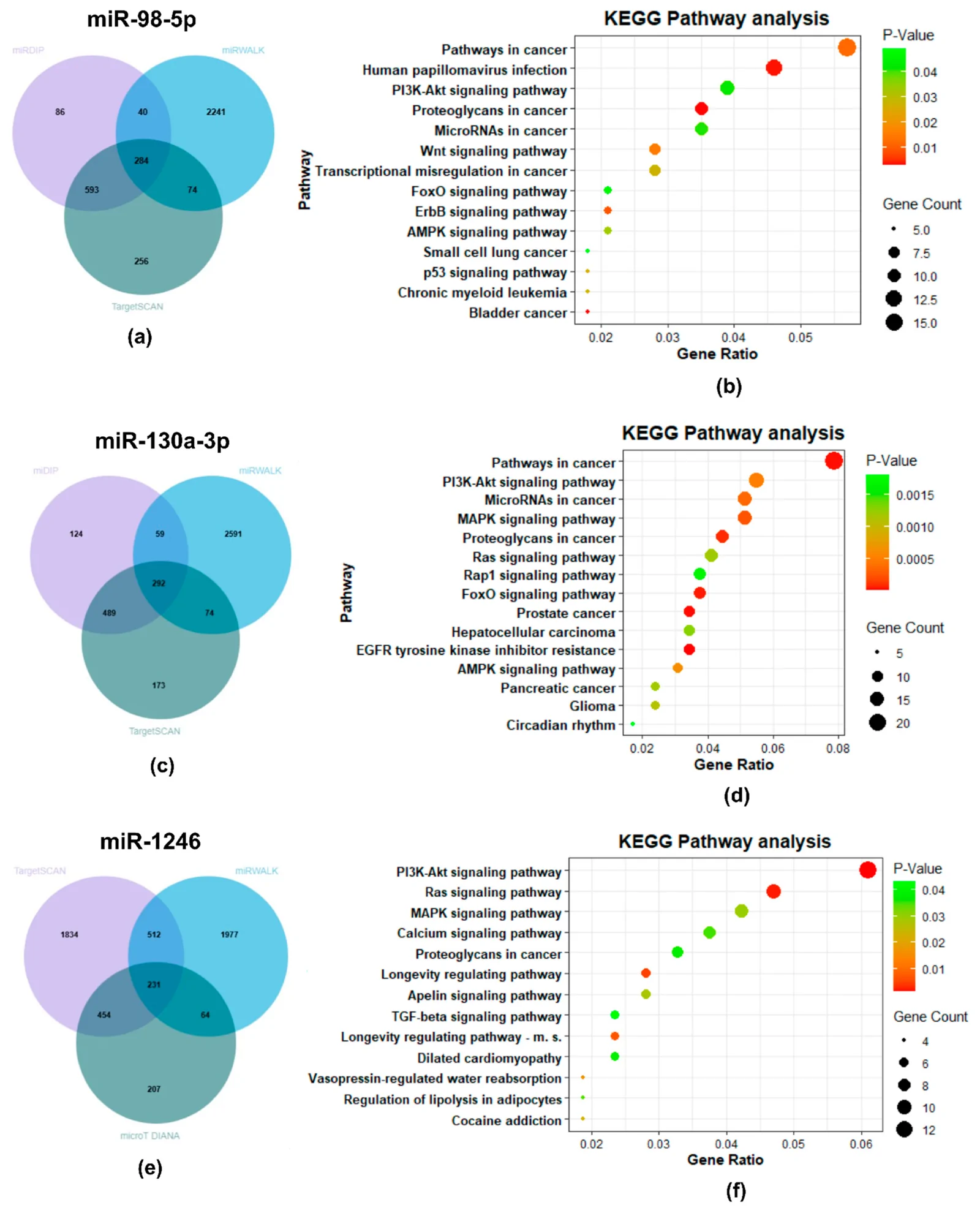
Target gene prediction and KEGG pathway enrichment analysis. (**a**,**c**,**e**) Venn diagrams showing the overlap among the predicted target genes of the selected miRNAs identified using the mirDIP, miRWalk, and TargetScan databases (**b**,**d**,**f**). Dot plots showing the most significantly enriched KEGG pathways associated with the predicted target genes. Panels (**a**,**b**) correspond to miR-98-5p, (**c**,**d**) to miR-130a-3p, and (**e**,**f**) to miR-1246.

To preliminarily explore the regulatory network of the selected miRNAs, we performed a comprehensive in silico analysis to identify predicted target genes followed by Gene Ontology (GO) and KEGG pathway enrichment analysis. For miR-98-5p, a total of 284 target genes were identified among the three prediction databases (Figure 7a).

Among the most significant terms in KEGG pathway analysis, interesting cancer-related pathways were found, such as ‘pathways in cancer’, ‘proteoglycan in cancer’, ‘microRNA in cancer’, and ‘transcriptional misregulation in cancer’. Additionally, key signalling pathways involved in tumorigenesis were identified, such as the PI3K-Akt, FoxO, and ErbB pathways (Figure 7b). Similarly, 292 predicted targets were identified for miR-130a-3p (Figure 7c). KEGG pathway analysis showed ‘pathways in cancer’ as the most relevant enriched term with 23 genes, followed by ‘PI3K-Akt signaling pathway’ and ‘microRNAs in cancer’. Interestingly, the analysis outlined the ‘hepatocellular carcinoma’ term with 10 genes (Figure 7d). For miR-1246, 231 common target genes were identified among the three prediction databases (Figure 7e). KEGG pathway analysis highlighted pathways associated with intracellular signal transduction whose dysregulation is a hallmark of cancer, including ‘PI3K-Akt signaling pathway’, ‘Ras signaling pathway’, ‘MAPK signaling pathway’, ‘Apelin signaling pathway’, and ‘TGF-beta signaling pathway’ (Figure 7f). Functional annotation of the same candidate genes revealed that miR-98-5p targets were enriched in biological processes related to cell cycle progression (e.g., ‘G1/S transition of mitotic cell cycle’, ‘Wnt signaling pathway’, and ‘cell division’) as well as regulation of transcription and translation (‘regulation of transcription by RNA polymerase II’, ‘negative regulation of translation’, and ‘negative regulation of transcription by RNA polymerase II’). Similarly, miR-130a-3p targets were related to transcriptional regulation and protein stability (‘positive regulation of transcription by RNA polymerase II’, ‘positive regulation of DNA-templated transcription’, and ‘protein stabilization’). Finally, for miR-1246, we found biological processes related to intracellular signal transduction driven by protein kinases (e.g., ‘cell surface receptor protein serine/threonine kinase signaling pathway’, ‘peptidyl-serine phosphorylation’). The top 10 terms for “biological process”, “cellular component”, and “molecular function” of the predictive targets are reported in Figure S5.

## 4. Discussion

To characterize the miRNA cargo of plasma-derived EVs, we performed exploratory miRNA-seq analysis in a discovery cohort including healthy subjects, patients with liver cirrhosis, and patients with hepatocellular carcinoma. Although our main interest was the comparison of miRNA levels between high-risk patients with cirrhosis and patients with HCC, we included HS because physiological miRNA levels may provide informative differences when compared with patients affected by hepatic diseases, such as chronic viral hepatitis or other conditions predisposing to cirrhosis. The membrane-affinity method that we used to purify RNA is not based on vesicle size, cellular origin, or the presence of a specific surface epitope and is intended to capture a broad spectrum of extracellular vesicles. However, because no characterization was performed, the isolated material should be referred to as an EV-enriched RNA fraction rather than a purified EV population. Among all the miRNAs detected in all the cohort groups, miR-98-5p, miR-130a-3p, and miR-1246 emerged as the most dysregulated across the different comparisons. Their discriminatory value therefore appears to rely on quantitative differences rather than on their mere presence or absence, an important characteristic for biomarkers intended for molecular monitoring in patients. Previous studies have described miR-98-5p and miR-130a-3p as showing cancer-type-specific expression trends [35,36,37,38,39]. miR-1246 has consistently been described as an oncomiR promoting proliferation, invasiveness, and metastatic capability [40]. In HCC, miR-98-5p is generally considered a tumor-suppressor miRNA; it is downregulated in tumor tissue and serum [41,42], and its ectopic upregulation restrains proliferation and invasion. miR-130a-3p has been reported to exert a tumor-suppressive role in HCC; it targets Smad4, and its reduced expression has been associated with a more aggressive tumor phenotype [43]. Conversely, miR-1246 is widely reported as an oncomiR in HCC [44]. Furthermore, our in silico analyses predicted that the three miRNAs converge on key oncogenic pathways, including PI3K-Akt, MAPK, Ras, and transcriptional regulation, suggesting their potential involvement in regulatory networks relevant to HCC biology. Collectively, these findings support the relevance of the selected miRNAs and justify the assessment of their levels in different types of biological samples of HCC, cirrhotic patients, and healthy subjects at both tissue and liquid biopsy levels to better understand their role in HCC. Previous studies investigating HCC tissues have similarly highlighted the diagnostic and prognostic relevance of specific miRNAs, reinforcing the rationale for evaluating miRNAs dysregulated across different biological matrices [45].

In plasma EVs, ddPCR analysis of miR-98-5p, miR-130a-3p, and miR-1246 in an independent and larger testing cohort showed a trend that partially overlapped with the miRNA-seq results. Specifically, miRNA-seq and ddPCR results were comparable in 5 out of 9 comparison classes (Table S8). This partial concordance may be explained by the limited size of the discovery cohort, as well as the fact that the subjects included in the testing cohort differed from those included in the discovery cohort [46]. However, the primary purpose of miRNA sequencing of plasma-derived EVs was to provide a global overview of their miRNA content and guide the prioritization of candidates for subsequent investigation. Our aim was to assess and compare the levels of selected miRNAs across different biological matrices from the same patients in a larger cohort distinct from the discovery cohort. Candidate selection was supported by published evidence proving the involvement of the three miRNAs in cancer in general and HCC in particular, together with our bioinformatic analyses. Accordingly, the sequencing data served as an exploratory, hypothesis-generating resource, and perfect concordance with subsequent measurements was not assumed.

In the testing cohort, the most relevant finding was significantly higher levels of miR-1246 in EVs of HCC patients compared with EVs of cirrhotic patients. In this context, comparing these two groups of patients is particularly relevant for introducing a candidate indicator of early detection in high-risk populations, as more than 80% of HCC cases arise in cirrhotic liver [1]. ROC curve analysis showed that miR-1246 has a good ability to distinguish patients with HCC from those with liver cirrhosis, suggesting its potential role as an early diagnostic biomolecule. Furthermore, in plasma-derived EVs of HCC patients, higher levels of miR-98-5p were associated with prolonged DFS, whereas elevated EV-associated miR-1246 levels characterized patients without relapses, suggesting its role as a candidate prognostic molecular indicator.

In plasma, higher circulating levels of miR-98-5p, miR-130a-3p, and miR-1246 were observed in HCC compared with LC with good diagnostic performance, as determined by ROC analysis. Increased miR-130a-3p and miR-1246 levels were associated with prolonged OS, whereas higher plasma miR-98-5p levels were observed in patients without relapses. Interestingly, higher miR-130a-3p plasma levels were associated with less aggressive histological features, suggesting a potential association with more favorable disease biology. Notably, miR-1246 displayed the same trend in both plasma fraction and plasma-derived EVs, supporting the maintenance of the same regulation across different circulating compartments. Moreover, the upregulation of plasma miR-1246 in HCC compared with HS is consistent with findings previously reported by Moshiri et al. [47].

To investigate whether circulating alterations were reflected at the tissue level, we quantified miRNA expression in paired tumoral and adjacent non-tumoral tissues from biopsies of patients with HCC. miR-98-5p was significantly upregulated in HCC compared with PT tissue, whereas miR-130a-3p showed the opposite trend. In contrast, miR-1246 displayed comparable expression levels in PT and HCC. These findings suggest that at least partially the detected circulating miRNAs may originate from hepatic tissue, although mechanistic insights were beyond the scope of this study.

A major strength of our study lies in the comprehensive profiling of miR-98-5p, miR-130a-3p, and miR-1246 across multiple circulating and tissue-derived matrices. This multi-matrix approach provides a broader and more integrated view of miRNA modulation in HCC. The assessment of their circulating amount both at EVs and cell-free plasmatic levels supports their possible role as biomolecules with potential significance for early diagnosis and stratification. Early diagnosis relies on distinguishing LC from HCC and is particularly relevant in cirrhotic patients, in whom early detection and molecular surveillance have the greatest clinical impact. In contrast, prognostic analyses stratify individuals within the HCC population according to clinical or molecular characteristics. In this light, circulating miRNAs have gained attention due to their stability and suitability for non-invasive longitudinal molecular monitoring. For example, miR-221 has been reported to be upregulated in the serum of patients with HCC. Furthermore, elevated miR-221 levels have been associated with a poorer prognosis [48]. Similarly, circulating serum miR-222 has been reported to be significantly upregulated in patients with HCC compared with patients with chronic hepatitis C and liver cirrhosis, supporting its potential as a non-invasive biomarker of disease progression [11]. miR-1246 consistently emerged as the most informative candidate diagnostic biomarker in EVs and in the plasma fraction, while miR-98-5p and miR-130a-3p are informative in the plasma fraction only. The combination of miR-1246 levels with circulating plasma levels of miR-98-5p, miR-130a-3p or both did not meaningfully improve diagnostic performance, suggesting miR-1246 captures the dominant biological signal distinguishing HCC from LC in our cohort.

The relatively small cohort size is a limitation of the present work. Although the investigation used separate discovery and testing cohorts, the testing cohort included 29 patients with HCC, 30 patients with liver cirrhosis, and 20 healthy subjects. Consequently, the estimated diagnostic performance, including AUC, sensitivity, and specificity, may be imprecise and potentially optimistic, particularly because the optimal cutoffs were selected and evaluated in the same testing cohort. The prognostic analyses are further limited by the small number of patients and outcome events, median-based dichotomization, and multiple comparisons, which increase the risk of unstable or chance associations. Therefore, the diagnostic and prognostic findings should be considered preliminary and hypothesis-generating. Validation in a larger, independent, prospectively enrolled clinical cohort using prespecified assays and decision thresholds is required before these miRNAs can be considered applicable translational biomarkers.

Sequencing and ddPCR findings were concordant in direction in five of nine pairwise comparisons. Therefore, the initial sequencing results were only partially replicated in the independent testing cohort. Although differences between cohorts and platforms used may have contributed, the discordance reduces confidence in the overall reproducibility of the results.

An additional limitation concerns the lack of EV characterization. The exoRNeasy workflow used in this study involved direct on-column lysis for RNA extraction; therefore, intact vesicles were not recovered for particle counting, size-distribution analysis, morphological examination, or EV-marker assessment. Moreover, the possible co-isolation of lipoproteins, soluble proteins, ribonucleoprotein complexes, or other non-vesicular RNA carriers was not evaluated. Accordingly, the measured miRNAs cannot be considered exclusively EV-associated, and the analyzed material is more appropriately interpreted as RNA recovered from a membrane-affinity-captured EV-enriched fraction. Future studies should include EV characterization and contaminant assessment.

## 5. Conclusions

In plasma-derived EVs, miR-98-5p and miR-130a-3p were lower in HCC and cirrhosis than in healthy subjects, but they did not distinguish HCC from cirrhosis; by contrast, EV-associated miR-1246 was higher in HCC than in either other group used in the cohort. In whole plasma, all three miRNAs were higher in HCC than in cirrhosis and healthy subjects. In paired tissues, miR-98-5p was higher and miR-130a-3p lower in HCC tissues than in peritumoral tissue, whereas miR-1246 did not differ significantly. Whole plasma contains both EV-associated and non-vesicular miRNA pools; therefore, differences between plasma and EV measurements may reflect selective vesicular loading, release in association with proteins or lipoproteins, contributions from non-hepatic cells, and differential clearance. As mentioned above, we explored the prognostic relevance of the selected miRNAs within the HCC cohort. To summarize, in HCC plasma-derived EVs, increased levels of miR-98-5p and miR-1246 characterized patients with longer DFS and patients without relapse, respectively. In the plasma fraction, high levels of miR-130a-3p and miR-1246 were associated with prolonged OS, whereas elevated miR-98-5p levels were found in patients without relapse. These findings suggest that EV isolation may not be strictly necessary for the diagnostic and prognostic use of the investigated miRNAs, as plasma-based measurements, particularly for miR-1246, provided comparable or even more informative results. For prognostic purposes, the determination of plasma levels of miR-98-5p and miR-130a-3p may be sufficient to predict tumor relapse and OS, respectively. Plasma analysis offers practical advantages by avoiding technically demanding and time-consuming procedures for EV isolation and molecular characterization. Plasma miR-1246 showed the strongest diagnostic performance among the three candidates evaluated for distinguishing HCC from liver cirrhosis in the present testing cohort. The associations observed for miR-98-5p, miR-130a-3p, and miR-1246 across circulating and tissue matrices provide preliminary evidence supporting their further investigation as candidate biomarkers. However, the diagnostic and prognostic findings remain exploratory and require confirmation in larger, independent, prospectively enrolled cohorts before clinical application.

## Figures and Tables

**Table 1:** Clinicopathological characteristics of the HCC patients included in the study.

		Discovery Cohort (*n* = 10)		Testing Cohort (*n* = 29)		*p*-Value *
Patient characteristics						
Age		42–80 (mean = 72)		58–80 (mean = 73)		0.721
Sex						1.000
Male		9	90%	26	89.66%	
Female		1	10%	3	10.34%	
Underlying liver disease						
Cirrhotic background						0.457
Absence of cirrhosis		3	30%	6	20.69%	
Grade 1 steatosis		4	40%	6	20.69%	
Grade 2 steatosis		/	/	1	3.45%	
Cirrhosis		3	30%	16	55.17%	
Hepatitis						0.109
Absence of hepatitis		6	60%	4	13.80%	
HBV		2	20%	9	31.03%	
HCV		2	20%	12	41.37%	
Toxic hepatitis		/	/	2	6.90%	
HBV with coinfection		/	/	2	6.90%	
AFP						1.000
<5 ng/mL		6	60%	20	68.96%	
≥5 ng/mL		3	30%	7	24.14%	
N/A		1	10%	2	6.90%	
Child-Pugh Score						
A		7	70%	28	96.55%	
N/A		3	30%	1	3.45%	
Tumor characteristics						
Grading						0.131
G2		9	90%	18	62.07%	
G3		1	10%	11	37.93%	
Number of nodules						1.000
Single		9	90%	24	82.76%	
Multiple		1	10%	5	17.24%	
Tumor diameter						
Median		3.5 cm		3.5 cm		
IQR		2.1–4.6 cm		2.5–5.9 cm		
Range		1.2–11.0 cm		1.2–15 cm		
BCLC stage						0.695
0		/	/	5	17.24%	
A		7	70%	20	68.97%	
B		/	/	4	13.79%	
N/A		3	30%	/	/	
MVI						0.020
Present		1	10%	14	51.72%	
Absent		8	80%	15	48.28%	
N/A		1	10%	/	/	
Follow-up and Clinical outcome						
Follow-up duration						
Median		35.87 months		34.42 months		
IQR		33.60–41.39 months		25.18–36.30 months		
Range		29.88–41.39 months		4.70–41.39 months		
Overall Survival						
Time of origin		Surgery date		Surgery date		
Death						
	DOD	0	/	7	24.14%	
	Alive/DOND	9	90%	22	75.86%	
	N/A	1	10%	/	/	
Disease-Free Survival						
Time of origin		Surgery date		Surgery date		
Relapse						0.273
	Present	3	30%	16	55.17%	
	Absent	7	70%	13	44.83%	

AFP: alpha-fetoprotein; BCLC: Barcelona Clinic Liver Cancer; MVI: Microvascular Invasion; DOD: died of disease; DOND: died of no disease. * Fisher’s exact test.

## Data Availability

All data generated or analyzed during this study are included in this article and its Supplementary Materials. The raw NGS sequencing data generated in this study are publicly available in the NCBI Sequence Read Archive (SRA) under accession number [PRJNA1525574]. Further enquiries can be directed to the corresponding author.

## Supplementary Materials

The following supporting information can be downloaded at https://www.mdpi.com/article/10.3390/cancers18203379/s1, Figure S1: Principal Component Analysis (PCA) of plasma-derived EV miRNA profiles in the discovery cohort; Figure S2: Heatmap showing the miRNAs detected in EVs across the three comparison groups (HCC vs. LC, HCC vs. HS, and LC vs. HS); Figure S3: Receiver Operating Characteristic (ROC) curve analysis of plasma miRNAs for the discrimination of HCC arising in cirrhotic patients from cirrhotic patients without HCC; Figure S4: Receiver Operating Characteristic (ROC) curve analysis of plasma miRNAs for discriminating early-stage HCC arising in cirrhotic patients from cirrhotic patients without HCC; Figure S5: GO enrichment analysis on the target genes of the selected miRNAs; Table S1: Clinicopathological characteristics of HCC patients with underlying cirrhosis included in the study; Table S2: Characteristics of HS and LC included in the study; Table S3: miRNAs differentially expressed in more than one comparison; Table S4: Summary of miRNA levels across plasma-derived EVs, plasma, and liver tissue detected by ddPCR; Table S5: Diagnostic performance of plasma miR-98-5p, miR-130a-3p, and miR-1246 in HCC vs. cirrhotic patients; Table S6: Diagnostic performance of plasma miR-98-5p, miR-130a-3p, and miR-1246 in HCC with cirrhosis vs. cirrhotic patients and early-stage HCC with cirrhosis vs. cirrhosis; Table S7: Non-significant associations between circulating miRNAs and clinicopathological features and prognosis in HCC patients; Table S8: Comparison of miRNA-seq (discovery cohort) and ddPCR (testing cohort) results for EV-associated miRNAs in plasma-derived extracellular vesicles.

## Abbreviations

The following abbreviations are used in this manuscript:

HCC	Hepatocellular Carcinoma
EVs	Extracellular Vesicles
miRNA	microRNA
ddPCR	droplet digital PCR
HBV	Hepatitis B Virus
HCV	Hepatitis C Virus
HDV	Hepatitis D Virus
AUC	Area Under the Curve
MASLD	Metabolic Dysfunction-Associated Steatotic Liver Diseas
NAFLD	Non-alcoholic Fatty Liver Disease
MASH	Metabolic Dysfunction-Associated Steatohepatits
NASH	Non-Alcoholic Steatohepatitis
ALD	Alcohol-related Liver Disease
EASL	European Association for the Study of the Liver
AASLD	American Association for the Study of Liver Disease
US	Ultrasound
AFP	Alpha-Fetoprotein
MRI	Magnetic Resonance Imaging
CT	Computed Tomography
CEUS	Contrast-Enhanced Ultrasound
ncRNAs	non-coding RNAs
NSCLC	Non-Small Cell Lung Cancer
OS	Overall Survival
DFS	Disease-Free Survival
MVBs	Multivesicular bodies
CRC	Colorectal Cancer
HS	Healthy Subjects
LC	Liver Cirrhosis patients
LNA	Locked Nucleic Acids
NGS	Next-Generation Sequencing
PCA	Principal Component Analysis
FDR	False Discovery Rate
BH	Benjamini–Hochberg
log_2_FC	log_2_ Fold Change
ROC	Receiver Operating Characteristic
CI	Confidence Interval
MVI	Microvascular Invasion
SEM	Standard Error of the Mean
PT	Peritumoral Tissue
GO	Gene Ontology
KEGG	Kyoto Encyclopedia of Genes and Genomes
AFP-L3	Lens culinaris agglutinin-reactive alpha-fetoprotein
DCP	Des-γ-Carboxy Prothrombin
GPC3	Glypican-3
