# Supplementary material for: miR-98-5p, miR-130a-3p, and miR-1246 Profiling in Tissue and Liquid Biopsies of Patients with Hepatocellular Carcinoma
Source: Cancers (Basel). 2026 Oct 19;18(20):3379. doi: 10.3390/cancers18203379 (PMC13702418; doi:10.3390/cancers18203379)
Supplement: Supplementary file 1 [file cancers-18-03379-s001.zip › Table S8.pdf]

Table S8. Comparison of miRNA-seq (discovery cohort) and ddPCR (testing cohort) results for EV-associated miRNAs in plasma-derived extracellular vesicles.

	**LC vs HS**				**HCC vs HS**				**HCC vs LC**			
	**miRNA-seq**		**ddPCR**		**miRNA-seq**		**ddPCR**		**miRNA-seq**		**ddPCR**	
	**log_2FC**	**padj**	**Trend**	**p**	**log_2FC**	**padj**	**Trend**	**p**	**log_2FC**	**padj**	**Trend**	**p**
miR-98-5p	-5.57	ns	Down	**	0.77	ns	Down	**	6.34	*	Equal	ns
miR-130a-3p	-0.26	ns	Down	*	-7.67	****	Down	*	-7.41	***	Down	ns
miR-1246	2.76	****	Equal	ns	2.55	****	Up	**	-0.21	ns	Up	**

HS: healthy subjects; LC: cirrhotic patients; HCC: HCC patients; miRNA-seq: miRNA sequencing; ddPCR: droplet digital PCR; log_2FC: log_2 Fold Change; p: p-value; padj: adjusted p-value.
