# Supplementary material for: miR-98-5p, miR-130a-3p, and miR-1246 Profiling in Tissue and Liquid Biopsies of Patients with Hepatocellular Carcinoma
Source: Cancers (Basel). 2026 Oct 19;18(20):3379. doi: 10.3390/cancers18203379 (PMC13702418; doi:10.3390/cancers18203379)
Supplement: Supplementary file 1 [file cancers-18-03379-s001.zip › Table S1.pdf]

Table S1. Clinicopathological characteristics of HCC patients with underlying cirrhosis included in the study.

			Testing cohort (n = 16)	
**Patient characteristics**				
Age			58 – 79 (mean = 72)	
Sex				
	Male		13	81.25%
	Female		3	18.75%
**Underlying liver disease**				
Cirrhotic background				
	Cirrhosis		16	100%
Hepatitis				
	HBV		4	25%
	HCV		8	50%
	Toxic hepatitis		2	12.50%
	HBV with coinfection		2	12.50%
AFP				
	< 5 ng/mL		10	62.50%
	≥ 5 ng/mL		6	37.50%
Child-Pugh Score				
	A		16%	100%
**Tumor characteristics**				
Grading				
	G2		11	68.75%
	G3		5	31.25%
Number of nodules				
	Single		12	75%
	Multiple		4	25%
Tumor diameter				
	Median		2.85 cm	
	IQR		2.15 – 4.10 cm	
	Range		1.30 – 10.50 cm	
BCLC stage				
	0		4	25%
	A		9	56.25%
	B		3	18.75%
MVI				
	Present		8	50%
	Absent		8	50%
**Follow-up and Clinical outcome**				
Follow-up duration				
	Median		34.42 months	
	IQR		25.18 – 36.30 months	
	Range		4.70 – 41.39 months	
Overall Survival				
	Time of origin		Surgery date	
	Death			
		DOD	3	18.75%
		Alive/DOND	13	81.25%
Disease-Free Survival				
	Time of origin			
	Relapse			
		Present	9	56.25%
		Absent	7	43.75%

AFP: alpha-fetoprotein; MVI: Microvascular Invasion; DOD: Died of Disease; DOND: Died Of No Disease.
