# Supplementary material for: miR-98-5p, miR-130a-3p, and miR-1246 Profiling in Tissue and Liquid Biopsies of Patients with Hepatocellular Carcinoma
Source: Cancers (Basel). 2026 Oct 19;18(20):3379. doi: 10.3390/cancers18203379 (PMC13702418; doi:10.3390/cancers18203379)
Supplement: Supplementary file 1 [file cancers-18-03379-s001.zip › Table S2.pdf]

Table S2. Characteristics of HS and LC included in the study.

	HS				LC			
	Discovery cohort (n = 10)		Testing cohort (n = 20)		Discovery cohort (n = 10)		Testing cohort (n = 30)	
Age	34 – 51 (mean 42)		30 – 62 (mean 46)		36 – 75 (mean 61)		43 – 82 (mean 66)	
Sex								
Male	6	60%	6	30%	8	80%	21	70%
Female	4	40%	12	60%	2	20%	9	30%
N/A	/	/	2	10%	/	/	/	/
Child-Pugh score	NED		NED		B – C		B – C	
Etiology								
Viral (HBV/HCV/HDV)					1	10%	4	13.33%
Metabolic					/	/	1	3.33%
Alcohol-related	NED		NED		1	10%	4	13.33%
Multifactorial					6	60%	16	53.33%
N/A					2	20%	5	16.68%

HS: Healthy Subjects; LC: patients with liver cirrhosis; NED: No Evidence of Disease.
