# Supplementary material for: miR-98-5p, miR-130a-3p, and miR-1246 Profiling in Tissue and Liquid Biopsies of Patients with Hepatocellular Carcinoma
Source: Cancers (Basel). 2026 Oct 19;18(20):3379. doi: 10.3390/cancers18203379 (PMC13702418; doi:10.3390/cancers18203379)
Supplement: Supplementary file 1 [file cancers-18-03379-s001.zip › Table S3.pdf]

Table S3. miRNAs differentially expressed in more than one comparison.

		LC vs HS			HCC vs HS			HCC vs LC		
**Target**	**Mean counts**	**log_2FC**	**p**	**padj**	**log_2FC**	**p**	**padj**	**log_2FC**	**p**	**padj**
**miR-130a-3p**	39.02	-0.26	ns	ns	-7.67	****	****	-7.41	****	***
miR-221-3p	7.62	-2.45	ns	ns	-5.91	***	**	-3.45	*	ns
miR-98-5p	11.22	-5.57	**	ns	0.77	ns	ns	6.34	**	*
miR-12136	159.14	-0-91	ns	ns	0.82	ns	ns	1.72	***	**
miR-142-5p	48.53	-1.15	ns	ns	0.67	ns	ns	1.83	**	*
let-7a-5p/7c-5p	616.08	-1.10	****	***	-0.98	****	**	0.11	ns	ns
**let-7g-5p**	68.76	-2.05	****	****	-0.63	ns	ns	1.43	**	*
**miR-486-3p**	77.94	1.18	**	*	-0.51	ns	ns	-1.69	****	**
miR-7704	192.34	1.02	**	*	0.57	ns	ns	-0.45	ns	ns
**miR-1246**	702.16	2.76	****	****	2.55	****	****	-0.21	ns	ns
miR-10396a-5p/ 10396b-5p	57.93	0.94	ns	ns	1.66	**	*	0.72	ns	ns
miR-8485	35.79	1.64	*	ns	2.18	**	*	0.54	ns	ns

Bolded miRNAs were significantly differentially expressed in at least two comparisons.

HS: healthy subjects; LC: cirrhotic patients; HCC: patients with HCC; log_2FC: log_2 Fold Change; p: p-value; padj: p-value adjusted. Significance levels are indicated as follows: p or padj < 0.05 (*); p or padj < 0.01 (**); p or padj < 0.001 (***); p or padj < 0.0001 (****).
