# Supplementary material for: miR-98-5p, miR-130a-3p, and miR-1246 Profiling in Tissue and Liquid Biopsies of Patients with Hepatocellular Carcinoma
Source: Cancers (Basel). 2026 Oct 19;18(20):3379. doi: 10.3390/cancers18203379 (PMC13702418; doi:10.3390/cancers18203379)
Supplement: Supplementary file 1 [file cancers-18-03379-s001.zip › Table S4.pdf]

Table S4. Summary of miRNA levels across plasma-derived EVs, plasma, and liver tissue detected by ddPCR.

Target	Plasma derived-EVs		Whole plasma		Liver tissue	
miR-98-5p	HCC vs LC	≈	HCC vs LC	↑****	HCC vs PT	↑*
	HCC vs HS	↓**	HCC vs HS	↑***		
	LC vs HS	↓**	LC vs HS	≈		
miR-130a-3p	HCC vs LC	≈	HCC vs LC	↑***	HCC vs PT	↓**
	HCC vs HS	↓*	HCC vs HS	↑**		
	LC vs HS	↓*	LC vs HS	↑		
miR-1246	HCC vs LC	↑**	HCC vs LC	↑****	HCC vs PT	≈
	HCC vs HS	↑**	HCC vs HS	↑****		
	LC vs HS	≈	LC vs HS	≈		

↑: increased; ↓: decreased; ≈: no significant difference; HS: healthy subjects; LC: cirrhotic patients; HCC: patients with HCC; PT: peritumoral tissue; HCC: tumoral tissue.
