# Supplementary material for: miR-98-5p, miR-130a-3p, and miR-1246 Profiling in Tissue and Liquid Biopsies of Patients with Hepatocellular Carcinoma
Source: Cancers (Basel). 2026 Oct 19;18(20):3379. doi: 10.3390/cancers18203379 (PMC13702418; doi:10.3390/cancers18203379)
Supplement: Supplementary file 1 [file cancers-18-03379-s001.zip › Table S5.pdf]

Table S5. Diagnostic performance of plasma miR-98-5p, miR-130a-3p, and miR-1246 in HCC vs cirrhotic patients.

Target	Sensitivity	Specificity	AUC	SE	95% CI	p-value
miR-98-5p	90%	77%	0.84	0.059	0.72 – 0-96	****
miR-130a-3p	79%	67%	0.75	0.066	0.62 – 0.88	***
miR-1246	83%	83%	0.87	0.047	0.78 – 0.96	****
miR-98-5p+miR-130a-3p	90%	77%	0.84	0.059	0.73 – 0.96	****
miR-98-5p+miR-1246	90%	77%	0.87	0.047	0.78 – 0.96	****
miR-130a-3p+miR-1246	79%	87%	0.86	0.049	0.76 – 0.95	****
miR-98-5p+miR-130a-3p+miR-1246	79%	83%	0.86	0.049	0.77 – 0.96	****

AUC: Area Under the Curve; SE: Standard Error; 95% CI: 95% Confidence Interval. Significance levels are indicated as follows: $p < 0.001$ (***); $p < 0.0001$ (****).
