# Supplementary material for: miR-98-5p, miR-130a-3p, and miR-1246 Profiling in Tissue and Liquid Biopsies of Patients with Hepatocellular Carcinoma
Source: Cancers (Basel). 2026 Oct 19;18(20):3379. doi: 10.3390/cancers18203379 (PMC13702418; doi:10.3390/cancers18203379)
Supplement: Supplementary file 1 [file cancers-18-03379-s001.zip › Table S6.pdf]

Table S6. Diagnostic performance of plasma miR-98-5p, miR-130a-3p, and miR-1246 in HCC with cirrhosis vs cirrhotic patients and early-stage HCC with cirrhosis vs cirrhosis.

Comparison	Target	Sensitivity	Specificity	AUC	SE	95% CI	p-value
HCC with cirrhosis vs LC	miR-98-5p	81%	87%	0.84	0.061	0.73 – 0.96	****
	miR-130a-3p	81%	67%	0.75	0.072	0.62 – 0.89	****
	miR-1246	81%	83%	0.87	0.052	0.76 – 0.97	***
Early-stage HCC with cirrhosis vs LC	miR-98-5p	85%	87%	0.83	0.065	0.71 – 0.96	****
	miR-130a-3p	92%	67%	0.79	0.068	0.66 – 0.93	****
	miR-1246	92%	70%	0.86	0.056	0.75 – 0.97	****

AUC: Area Under the Curve; SE: Standard Error; 95% CI: 95% Confidence Interval. Significance levels are indicated as follows: $p < 0.001$ (***); $p < 0.0001$ (****).
