# Supplementary material for: miR-98-5p, miR-130a-3p, and miR-1246 Profiling in Tissue and Liquid Biopsies of Patients with Hepatocellular Carcinoma
Source: Cancers (Basel). 2026 Oct 19;18(20):3379. doi: 10.3390/cancers18203379 (PMC13702418; doi:10.3390/cancers18203379)
Supplement: Supplementary file 1 [file cancers-18-03379-s001.zip › Table S7.pdf]

Table S7. Non-significant associations between circulating miRNAs and clinicopathological features and prognosis in HCC patients.

miRNA	Analysis	Comparison	p-value
**miR-98-5p: Plasma**	DFS	High *vs* Low	ns
	OS	High *vs* Low	ns
	Cirrhotic Background (absence of cirrhosis, grade 1 steatosis, grade 2 steatosis, and cirrhosis)	Kruskal-Wallis test	ns
	Hepatitis (absence of hepatitis, HBV, HCV, toxic hepatitis, and HBV with coinfection)	Kruskal-Wallis test	ns
	AFP (< 5 ng/mL, ≥ 5 ng/mL)	Mann-Whitney test	ns
	Grading (G2, G3)	Mann-Whitney test	ns
	Number of nodules (Single, Multiple)	Mann-Whitney test	ns
	MVI (Present, Absent)	Mann-Whitney test	ns
**miR-98-5p: Plasma-derived EVs**	OS	High *vs* Low	ns
	Cirrhotic Background (absence of cirrhosis, grade 1 steatosis, grade 2 steatosis, and cirrhosis)	Kruskal-Wallis test	ns
	Hepatitis (absence of hepatitis, HBV, HCV, toxic hepatitis, and HBV with coinfection)	Kruskal-Wallis test	ns
	AFP (< 5 ng/mL, ≥ 5 ng/mL)	Mann-Whitney test	ns
	Grading (G2, G3)	Mann-Whitney test	ns
	Number of nodules (Single, Multiple)	Mann-Whitney test	ns
	MVI (Present, Absent)	Mann-Whitney test	ns
	Relapse (Present, Absent)	Mann-Whitney test	ns
**miR-130a-3p: Plasma**	DFS	High *vs* Low	ns
	Cirrhotic Background (absence of cirrhosis, grade 1 steatosis, grade 2 steatosis, and cirrhosis)	Kruskal-Wallis test	ns
	Hepatitis (absence of hepatitis, HBV, HCV, toxic hepatitis, and HBV with coinfection)	Kruskal-Wallis test	ns
	AFP	Mann-Whitney test	ns

	(< 5 ng/mL, ≥ 5 ng/mL)		
	Number of nodules (Single, Multiple)	Mann-Whitney test	ns
	MVI (Present, Absent)	Mann-Whitney test	ns
	Relapse (Present, Absent)	Mann-Whitney test	ns
**miR-130a-3p: Plasma-derived EVs**	DFS	High *vs* Low	ns
	OS	High *vs* Low	ns
	Cirrhotic Background (absence of cirrhosis, grade 1 steatosis, grade 2 steatosis, and cirrhosis)	Kruskal-Wallis test	ns
	Hepatitis (absence of hepatitis, HBV, HCV, toxic hepatitis, and HBV with coinfection)	Kruskal-Wallis test	ns
	AFP (< 5 ng/mL, ≥ 5 ng/mL)	Mann-Whitney test	ns
	Grading (G2, G3)	Mann-Whitney test	ns
	Number of nodules (Single, Multiple)	Mann-Whitney test	ns
	MVI (Present, Absent)	Mann-Whitney test	ns
	Relapse (Present, Absent)	Mann-Whitney test	ns
**miR-1246: Plasma**	DFS	High *vs* Low	ns
	Cirrhotic Background (absence of cirrhosis, grade 1 steatosis, grade 2 steatosis, and cirrhosis)	Kruskal-Wallis test	ns
	Hepatitis (absence of hepatitis, HBV, HCV, toxic hepatitis, and HBV with coinfection)	Kruskal-Wallis test	ns
	AFP (< 5 ng/mL, ≥ 5 ng/mL)	Mann-Whitney test	ns
	Grading (G2, G3)	Mann-Whitney test	ns
	Number of nodules (Single, Multiple)	Mann-Whitney test	ns
	MVI (Present, Absent)	Mann-Whitney test	ns
	Relapse (Present, Absent)	Mann-Whitney test	ns
**miR-1246: Plasma-derived EVs**	DFS	High *vs* Low	ns

OS	High *vs* Low	ns
Cirrhotic Background (absence of cirrhosis, grade 1 steatosis, grade 2 steatosis, and cirrhosis)	Kruskal-Wallis test	ns
Hepatitis (absence of hepatitis, HBV, HCV, toxic hepatitis, and HBV with coinfection)	Kruskal-Wallis test	ns
AFP (< 5 ng/mL, ≥ 5 ng/mL)	Mann-Whitney test	ns
Grading (G2, G3)	Mann-Whitney test	ns
Number of nodules (Single, Multiple)	Mann-Whitney test	ns
MVI (Present, Absent)	Mann-Whitney test	ns

OS: Overall Survival; DFS: Disease-Free Survival; AFP: alpha-fetoprotein; MVI: microvascular invasion.
